# COVID-19 vaccine hesitancy among medical and health science students attending Wolkite University in Ethiopia

**DOI:** 10.1371/journal.pone.0263081

**Published:** 2022-01-25

**Authors:** Ayenew Mose, Kassahun Haile, Abebe Timerga

**Affiliations:** 1 Department of Midwifery, College of Medicine and Health Science, Wolkite University, Wolkite, Ethiopia; 2 Department of Medical Laboratory Science, College of Medicine and Health Science, Wolkite University, Wolkite, Ethiopia; 3 Department of Biomedical Science, College of Medicine and Health Science, Wolkite University, Wolkite, Ethiopia; Bucharest University of Economic Studies, ROMANIA

## Abstract

**Background:**

Medical and health science students are among the frontline health care workers who are at high risk of acquiring COVID-19 infection during their clinical attachments and future career. As health care providers, they are expected to promote and administer the COVID-19 vaccine and counsel vaccine-hesitant patients. It is, therefore, imperative to assess COVID-19 vaccine hesitancy among medical and health science students. Thus, this study aimed to assess COVID-19 vaccine hesitancy and its associated factors among medical and health science students of Wolkite University.

**Method:**

An institutional-based cross-sectional study design was conducted among 420 medical and health science students attending Wolkite University from March 1 to 30, 2021. Simple random sampling technique was used to select study participants. Self-administered and structured questionnaires were used to collect data. Data were entered into Epi-Data version 4.2.0 and exported to SPSS version 23 software package for further analysis. Bivariable and multivariable analysis was used to identify associated factors. P values <0.05 result were considered as a statistically significant association.

**Results:**

The level of COVID-19 vaccine hesitancy was 41.2% (95% CI; 35.2%-50.4%). Student age ≤23 years were 1.9 times more likely vaccine hesitant [aOR = 1.94, 95% CI; 1.14–3.28], being female were 1.7 times more likely vaccine hesitant [aOR = 1.76, 95% CI; 1.14–2.72], resided in rural area were 1.6 times more likely vaccine hesitant [aOR = 1.63, 95% CI; 1.06–2.49], source of information from social media were 2.7 times more likely vaccine hesitant [aOR = 2.68, 95% CI; 1.58–4.54], and good practice to COVID-19 mitigation measures were 47% less likely vaccine hesitant [aOR = 0.53, 95% CI; 0.34–0.83] compared to their counterpart.

**Conclusions:**

COVID-19 vaccine hesitancy is found to be high. Therefore, students are advised to receive COVID-19 vaccine information from government lead mass media (i.e. television and radio), increase awareness and adherence to COVID-19 mitigation measures is recommended.

## Background

In 2019, the World Health Organization states that vaccine hesitancy is one of the top ten global health threats [1]. Vaccine hesitancy is defined as the delay in acceptance, reluctance, or refusal of vaccines despite the availability of vaccination services [2]. Worldwide, more than 90% of countries have encountered vaccine hesitancy, which can seriously jeopardize the successful implementation of vaccination campaigns [3].

Since the onset of the COVID-19 pandemic in early December 2019, in Wuhan city, China, infects 166, 352,007+ confirmed cases and 3,449,189+ deaths as of 23 May 2021 globally. On the African continent alone accounts 3,446,089+ confirmed cases and 85,964+ deaths [4, 5]. In Ethiopia, as of 23 May 2021, 269,194+ confirmed COVID-19 cases and 4,076+ deaths is recorded [6]. The proportions of health care workers COVID-19 infection is varied from 2.2% to 29% [7]. A systemic review showed that 15,2888 infections and 1,413 deaths have occurred among health care workers [8].

The first COVID-19 vaccines arrived in Ethiopia on 6 March 2021. By the end of 2021, Ethiopia has planned to vaccinate 20% of the population [9]. As of 23 May 2021, 1,655,244+ COVID-19 vaccines were administered in Ethiopia [6].

Vaccination is one of the promising strategies to combat the COVID-19 pandemic. However, vaccine hesitancy was reported globally among medical students. For instance, 46% of Egyptian medical students [10], 30.6% of Uganda medical students [11], 10.6% of Indian medical students [12], 13.6% of Italy university students [13], and 23% of Michigan medical students [14] were hesitant for COVID-19 vaccination.

Vaccine hesitancy is complex and influenced by multiple factors. Some of the factors associated with COVID-19 vaccine hesitancy are lack of confidence in the vaccine itself, lack of adequate information about the vaccine, misinformation from social media, conspiracy theories, and fear of side effects [2, 10, 11, 14].

According to the WHO prioritizing roadmap; frontline health care workers, elder people, and those who had medical illnesses are prioritized for COVID-19 vaccination [15]. Medical and health science students are among the frontline health care workers who are at high risk of acquiring COVID-19 during their clinical attachment. As health care providers, they are expected to promote and administer the COVID-19 vaccine and counsel vaccine-hesitant patients. It is, therefore, imperative to understand vaccine hesitancy in this group of population.

Several literatures were found regarding COVID-19 vaccine hesitancy among health care workers and the general population [16–20]. However, there is paucity of studies regarding COVID-19 vaccine hesitancy among medical and health science students in Ethiopia. Therefore, this study aimed to assess COVID-19 vaccine hesitancy and its associated factors among medical and health science students of Wolkite University.

## Method and materials

### Study design, area, and period

We conducted an institutional-based cross-sectional study design among medical and health science students of Wolkite University from March 1 to 30, 2021. Wolkite University is one of the third generation higher institutions that have been established in 2012 G.C. Wolkite University is located in Gurage zone, Southern Nation Nationalities Regional State, 158 km southwest of Addis Ababa. The University has contained seven colleges namely; the college of medicine and health science, computing and informatics, engineering and technology, natural and computational science, agricultural science, social science and humanities, and business and economics. The college of medicine and health science had 5 departments (i.e. medicine, public health, midwifery, nursing, and medical laboratory science).

### Source population and study population

All regular undergraduate students who were attending Wolkite University, College of Medicine and Health Science were considered as source population. All students who were selected using simple random sampling technique were considered as the study population. All undergraduate students who were found at Wolkite University, College of Medicine and Health Science during the study period were included.

### Sample size determination and sampling procedure

The required sample size for this study was determined using a single population proportion formula by considering the following assumptions; the proportion of COVID-19 vaccine hesitancy among medical students in Michigan (p = 23%) [14], a margin of error (d = 5%), at 95% CI (Z_α/2_ = 1.96), and 10% of the non-respondent rate. The final sample size was 420 for this study. In Wolkite University, college of medicine and health science, there are 5 departments (i.e, medicine, public health officer, medical laboratory sciences, midwifery, and nursing). Later on, the proportional allocation was used for each department and academic year. Finally, using a student attendance list, a simple random sampling technique was used to withdraw 420 study participants; accordingly, 122 from medicine, 65 from public health, 60 from midwifery, 124 from nursing, and 49 from medical laboratory science department were included.

### Data collection tools

The questionnaires were adapted after reviewing relevant literature regarding COVID-19 vaccine hesitancy [12, 14, 21–24]. Data collection tool was prepared in English language, as it is the medium of instruction in Ethiopian higher educational institutions. The questionnaire was designed to collect information regarding student socio-demographic characteristics (age, sex, residence, department, year, father and mother educational status), source of information about COVID-19 vaccine, knowledge about COVID-19 vaccine, attitude towards COVID-19 vaccine, practice towards COVID-19 mitigation measures, COVID-19 vaccine hesitancy, reasons of vaccine hesitancy, and vaccine acceptance.

### Data collection procedure and quality control

A total of 5 health professionals (3 BSc undergraduate data collectors and 2 MSc holder supervisors) were participated in the data collection process. One day theoretical and practical training was given for data collectors on the objective of the study and methods of data collection. Data collectors and supervisors were informed to follow the World Health Organization COVID-19 prevention protocols such as wearing a facemask, maintaining physical distancing, and using hand sanitizer during data collection time. Two supervisors with the principal investigator were closely following the data collection process throughout the data collection period. Pre-test were done on 21(5%) of post basic health science students. Self-administered, structured, and close-ended questionnaires were used to collect data.

### Study variables and measurements

COVID-19 vaccine hesitancy is defined as a refusal of accepting the COVID-19 vaccine despite the availability of the vaccination service [2]. In this study, we assessed COVID-19 vaccine hesitancy by asking a question, ‘‘Do you have an intention to be vaccinated against COVID-19 infection, if the COVID-19 vaccine is available right now?”. Those students who answered ‘Yes’ are codded as COVID-19 vaccine acceptance and those students who answered ‘No’ are coded as COVID-19 vaccine hesitancy [11]. Students who had scored less than 70% are coded as poor knowledge, and those students who had scored more than or equal to 70% of knowledge related items are codded as good knowledge [23]. Similarly, for attitude related items; those students who had scored less than 70% are coded as unfavourable attitude and those students who had scored greater than or equal to 70% coded as unfavourable attitude [23]. Regarding students practice towards COVID-19 mitigation measures, those students who had practiced more than or equal to 75% of COVID-19 mitigation measures related questions correctly were interpreted as good practice whereas those students who had responses less than 75% were interpreted as poor practice [21].

### Data analysis

All the questionnaires were checked manually for completeness, then entered into Epi-Data version 4.2.0 and exported to SPSS version 23 software package for analysis. Descriptive analysis results were presented in the form of tables, figures, and pie charts. Text using frequencies and summary statistics such as mean, standard deviation, and percentage were used. Internal consistency reliability of knowledge and attitude items were checked on 21 pre-test questionnaires and giving a result of Cronbach’s alpha (α = 0.81) and (α = 0.71) respectively. Bivariate logistic regression analysis was used to determine the association of each independent variable with the outcome variable by using binary logistic regression. The goodness of fit was tested by Hosmer-Lemeshow statistic and Omnibus tests give us a result of (0.87). The direction and strength of statistical association are measured by odds ratio with 95%CI. Adjusted odds ratio along with 95%CI is estimated to identify predictors for COVID-19 vaccine hesitancy by using multivariable analysis in the binary logistic regression model. In this study, P-value <0.05 was considered to declare a result as a statistically significant association.

### Ethical considerations

The ethical approval was obtained from Wolkite University, College of Medicine and Health Science ethical review board. The objective of the study was briefly explained to the study participants. Later on, informed and written consent was obtained from each study subject prior to the data collection process preceded. Study participants who were not willing to participate in the study have been given the right to refuse, withdraw their part in the study.

## Results

### Socio-demographic characteristics of study participants

A total of 420 students were involved in this study, which made a response rate of 100%. About 249 (59.3%) of study participants were male. The majority, 324 (77.1%) of students were found in the age group between 19 to 23 years and the mean age of students was 22.7±1.5 years. Concerning residence, 227 (54%) of students resided in a rural area. The majority, 92.6% of the study participants were single. About 198 (47.1%) and 203 (48.3%) of study participants mothers and fathers were completed primary education respectively. Regarding students department and academic year, 124 (29.5%) of study participants were from the nursing department, and 145 (34.5%) of study participants were 4^th^ year (Table 1).

**Table 1:** Socio-demographic characteristics of medical and health science students attending Wolkite University, Ethiopia, 2021 (n = 420).

Variables	Categories	Frequency	Percentage (%)
Gender	Female	171	40.7
	Male	249	59.3
Age	≤23	324	77.1
	>24	96	22.9
Residence	Rural	227	54.0
	Urban	193	46.0
Marital status	Single	389	92.6
	Married /engaged	31	7.4
Mother educational status	No formal education	121	28.8
	Primary education	198	47.1
	Secondary and above	101	24.1
Father educational status	No formal education	92	21.9
	Primary	203	48.3
	Secondary and above	125	29.8
Department	Medicine	122	29.0
	Public health	65	15.5
	Midwifery	60	14.3
	Nursing	124	29.5
	Medical laboratory	49	11.7
Academic year	2nd year	108	25.7
	3rd year	132	31.4
	4th year	145	34.5
	5th year	35	8.3

### Source of information about COVID-19 vaccine

**Fig 1:**
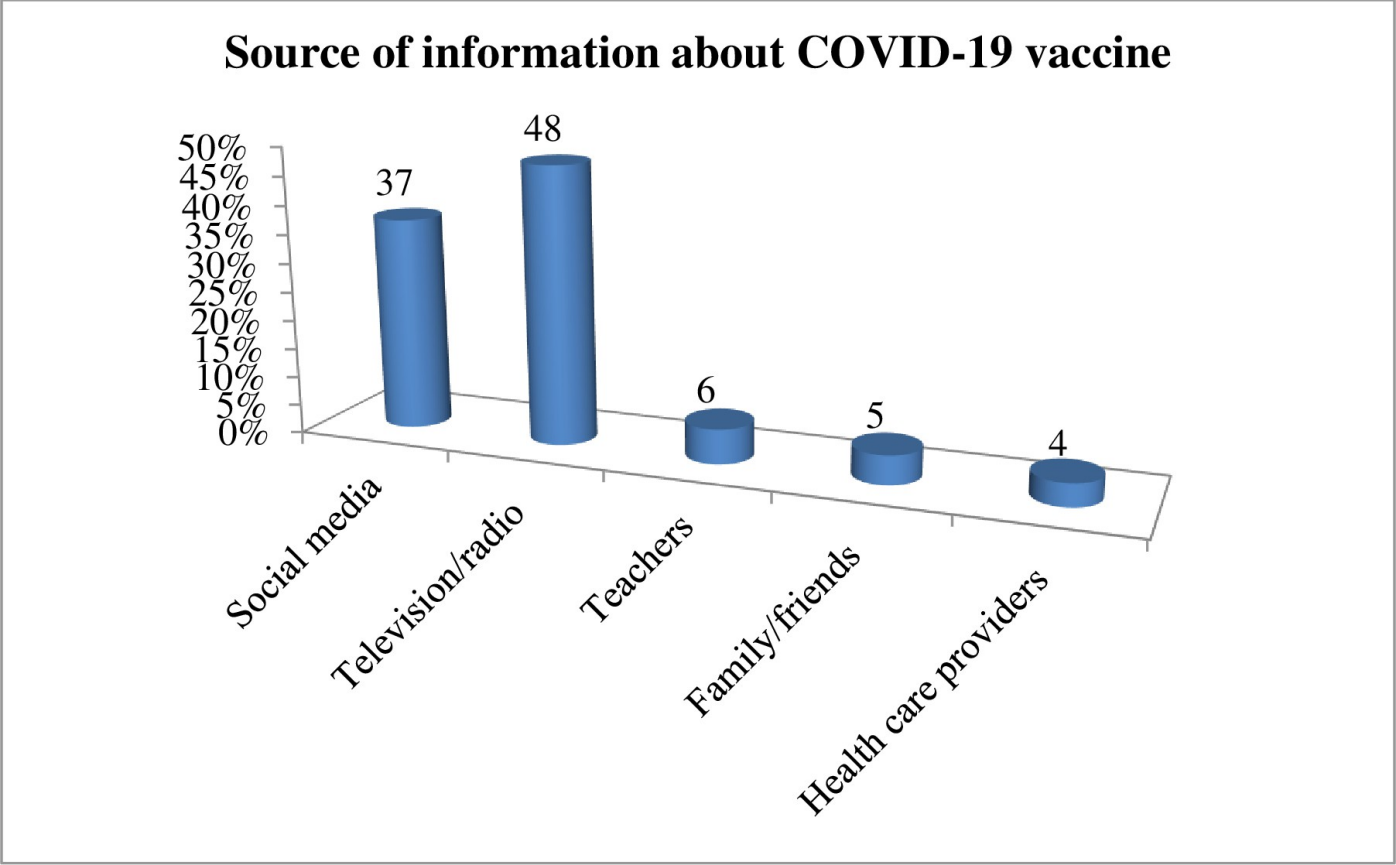
Source of information about COVID-19 vaccine among medical and health science students attending Wolkite University, Ethiopia, 2021 (n = 420).

All of the study participants (420) had information about the COVID-19 vaccine. The majority, 202 (48%) of study participants were heard about the COVID-19 vaccine from television/radio and 155 (37%) of study participants were heard from social media (i.e. Facebook and Telegram) (Fig 1).

### Knowledge about COVID-19 vaccine

This study found that 394 (94%) of study participants know that COVID-19 is currently available in Ethiopia, and 310 (73.8%) of study participants believe that the COVID-19 vaccine is effective to control the pandemic. The majority, 302 (71.8%) of study participants responded that health care workers should receive vaccines firstly compared to the general population and 352 (83.8%) of study participants responded that COVID-19 vaccination is important for community health and overall for public health. Two hundred ninety-two (69.5%) and 219 (52.1%) of study participants responded that COVID-19 vaccination does not cause autoimmune diseases and allergic reactions respectively. About 297 (70.7%) of study participants responded that COVID-19 vaccination can reduce the severity of the diseases. Overall, 330 (78.6%) of study participants have good knowledge about the COVID-19 vaccine while 90 (21.4%) of study participants have poor knowledge about the COVID-19 vaccine (Table 2).

**Table 2:** Knowledge about COVID-19 vaccine among medical and health science students attending Wolkite University, Ethiopia, 2021 (n = 420).

Variables	Categories	Frequency	Percentage (%)
COVID-19 vaccine is available in Ethiopia	Yes	395	94.0
	No	20	4.8
	I don’t know	5	1.2
COVID-19 vaccine is effective	Yes	310	73.8
	No	94	22.4
	I don’t know	16	3.8
COVID-19 vaccine should be given for HCWs firstly	Yes	302	71.9
	No	93	22.1
	I don’t know	25	6.0
COVID-19 vaccination is important for overall public health	Yes	352	83.8
	No	32	7.6
	I don’t know	36	8.6
COVID-19 vaccination doesn’t cause autoimmune diseases	Yes	292	69.5
	No	79	18.8
	I don’t know	49	11.7
COVID-19 vaccination doesn’t cause allergic reaction	Yes	219	52.1
	No	176	41.9
	I don’t know	25	6.0
COVID-19 vaccination decrease severity of diseases	Yes	297	70.7
	No	85	20.2
	I don’t know	38	9.0
Knowledge	Good knowledge	330	78.6
	Poor knowledge	90	21.4

### Attitude towards COVID-19 vaccine

In this study, 394(91.2%) and 338(80.5%) of study participants agreed that COVID-19 vaccination should be mandatory for health care workers as well as for the general public respectively. Two-third, 281(66.1%) of study participants believe that the COVID-19 vaccine can reduce the spread of the virus in the community. More than two-fourth, 185(44%) of study participants would encourage their family, friends, and relatives to get vaccinated if it is once available. The overall attitude towards the COVID-19 vaccine was found 354 (84.5%) of study participants have a favourable attitude while only 66 (15.7%) have unfavourable attitude (Table 3).

**Table 3:** Attitude towards COVID-19 vaccine among medical and health science students attending Wolkite University, Ethiopia, 2021 (n = 420).

Variables	Categories	Frequency	Percentage (%)
COVID-19 vaccine should be made mandatory for the health care workers?	Agree	394	91.2
	Disagree	34	8.1
	I am not Shure	3	0.7
COVID-19 vaccination should be mandatory for the general public	Agree	338	80.5
	Disagree	53	12.6
	I am not Shure	29	6.9
I will take the COVID-19 vaccine; if it is available in the hospital right know	Agree	181	43.1
	Disagree	172	41.0
	I am not Shure	67	16.0
COVID-19 vaccine reduce the spread of the disease in the community	Agree	281	66.9
	Disagree	98	23.3
	I am not Shure	41	9.8
I will encourage my family/friends/relatives to get vaccinated?	Agree	185	44.0
	Disagree	228	54.3
	I am not Shure	7	1.7
COVID-19 vaccine is safe	Agree	290	69.0
	Disagree	77	18.3
	I am not Shure	53	12.6
COVID-19 vaccination reduces the severity of the disease?	Agree	348	82.9
	Disagree	62	14.8
	I am not Shure	10	2.4
Attitude	Favourable attitude	354	84.5
	Unfavourable attitude	66	15.7

### Practice of COVID-19 mitigation measures

The present study showed that 239(56.9%), 136(32.4%), 289(68.8%), 379(90.2%) of study participants practice frequent utilization of face masks, maintain physical distancing at least 2m, covered their mouth during coughing /sneezing/, and regular hand washing using water and soap for at least 20 seconds respectively. Overall, good practice towards COVID-19 mitigation measures was found to be 41% (95%, CI; 36.3%-45.1%) (Table 4).

**Table 4:** Practice of COVID-19 mitigation measures among medical and health science students attending Wolkite University, Ethiopia, 2021 (n = 420).

Variable	Categories	Frequency	Percentage (%)
Frequent utilization of face mask	Yes	239	56.9
	No	181	43.1
Maintained physical distancing at least 6 feet (2m)	Yes	136	32.4
	No	284	67.6
Covered your mouth during coughing /sneezing/	Yes	289	68.8
	No	131	31.2
Avoid greeting with handshaking, hanging/cheek kissing	Yes	342	81.4
	No	78	18.6
Frequent utilized alcohol-based hand sanitizer	Yes	341	81.2
	No	79	18.8
Regular hand washing using water and soap for at least 20 second	Yes	379	90.2
	No	41	9.8
Avoided touching face (eyes, nose, mouth)	Yes	237	56.4
	No	183	43.6
Clean and disinfecting frequently touched objects like phone	Yes	123	29.3
	No	297	70.7
Stay at home when having flu-like symptoms	Yes	160	38.1
	No	260	61.9
Avoid going unnecessarily to crowed place	Yes	113	26.9
	No	307	73.1
Avoid gathering with many people	Yes	243	57.9
	No	177	42.1
Avoid eating uncooked food	Yes	347	82.6
	No	73	17.4
Practice	Poor practice	248	59.0
	Good practice	172	41.0

### COVID-19 vaccine acceptance/hesitancy

**Fig 2:**
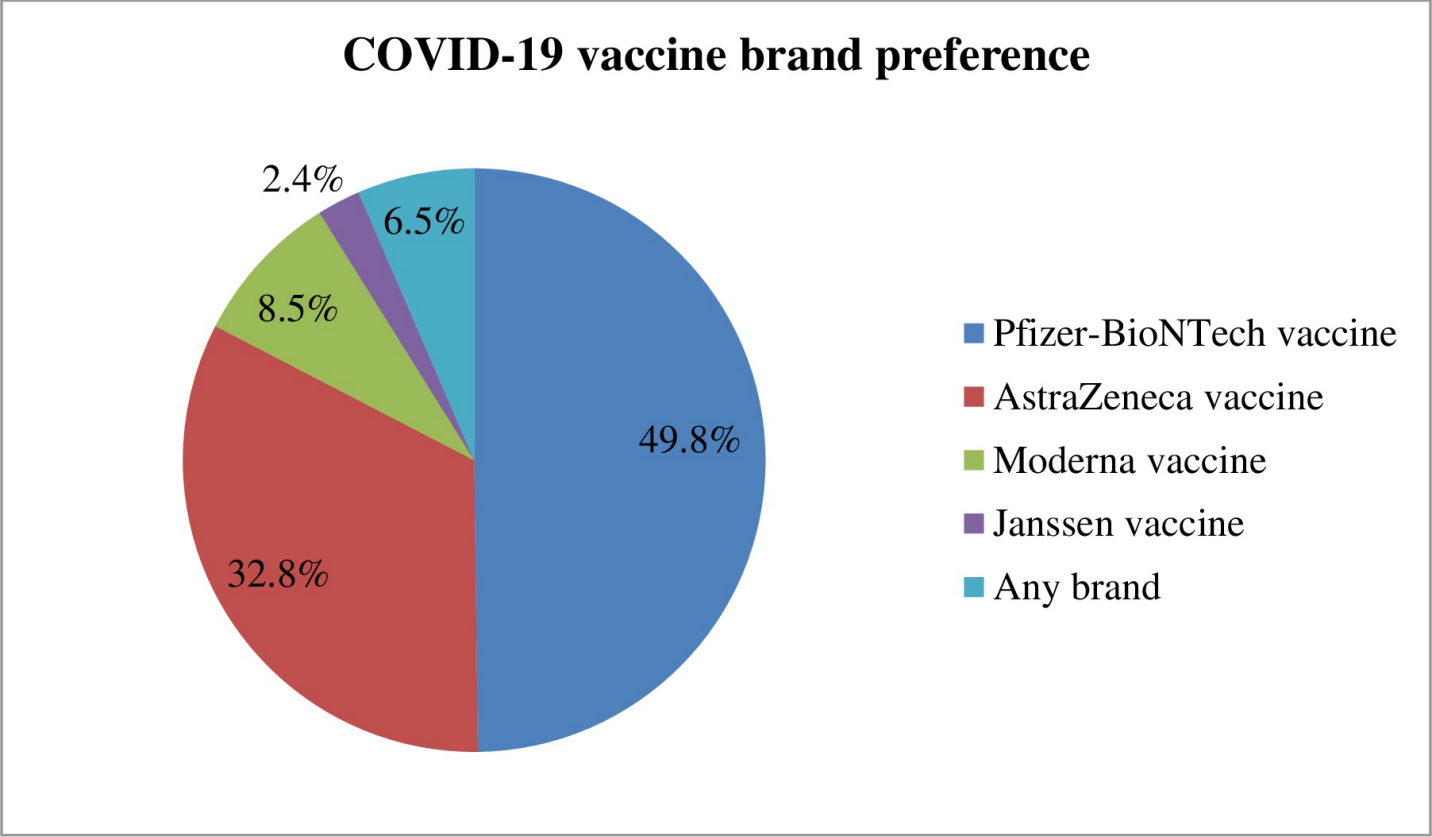
COVID-19 vaccine brand preference among medical and health science students attending Wolkite University, Ethiopia, 2021 (n = 247).

**Fig 3:**
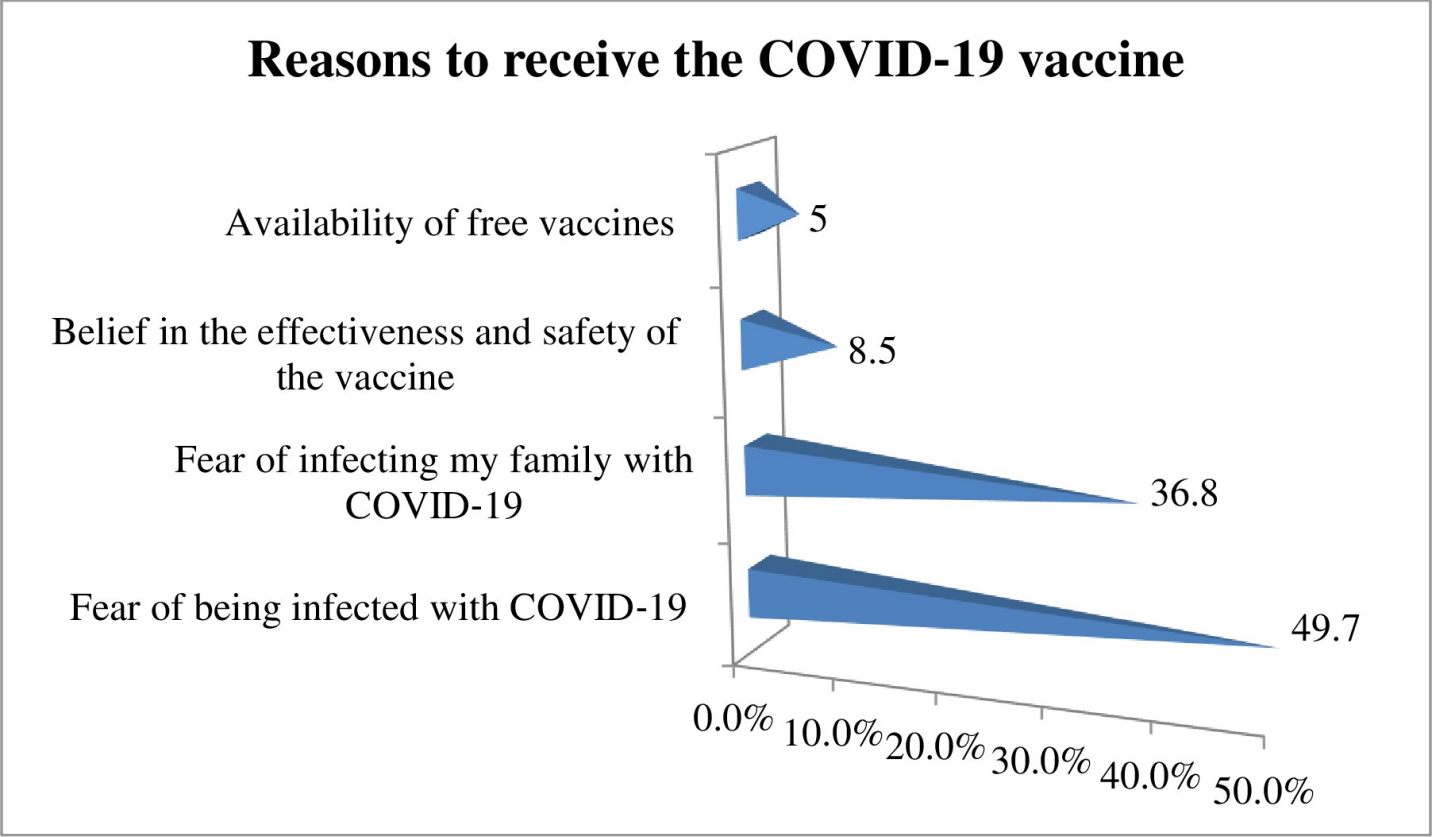
Reasons for willingness to receive COVID-19 vaccine among medical and health science students attending Wolkite University, Ethiopia, 2021 (n = 247).

**Fig 4:**
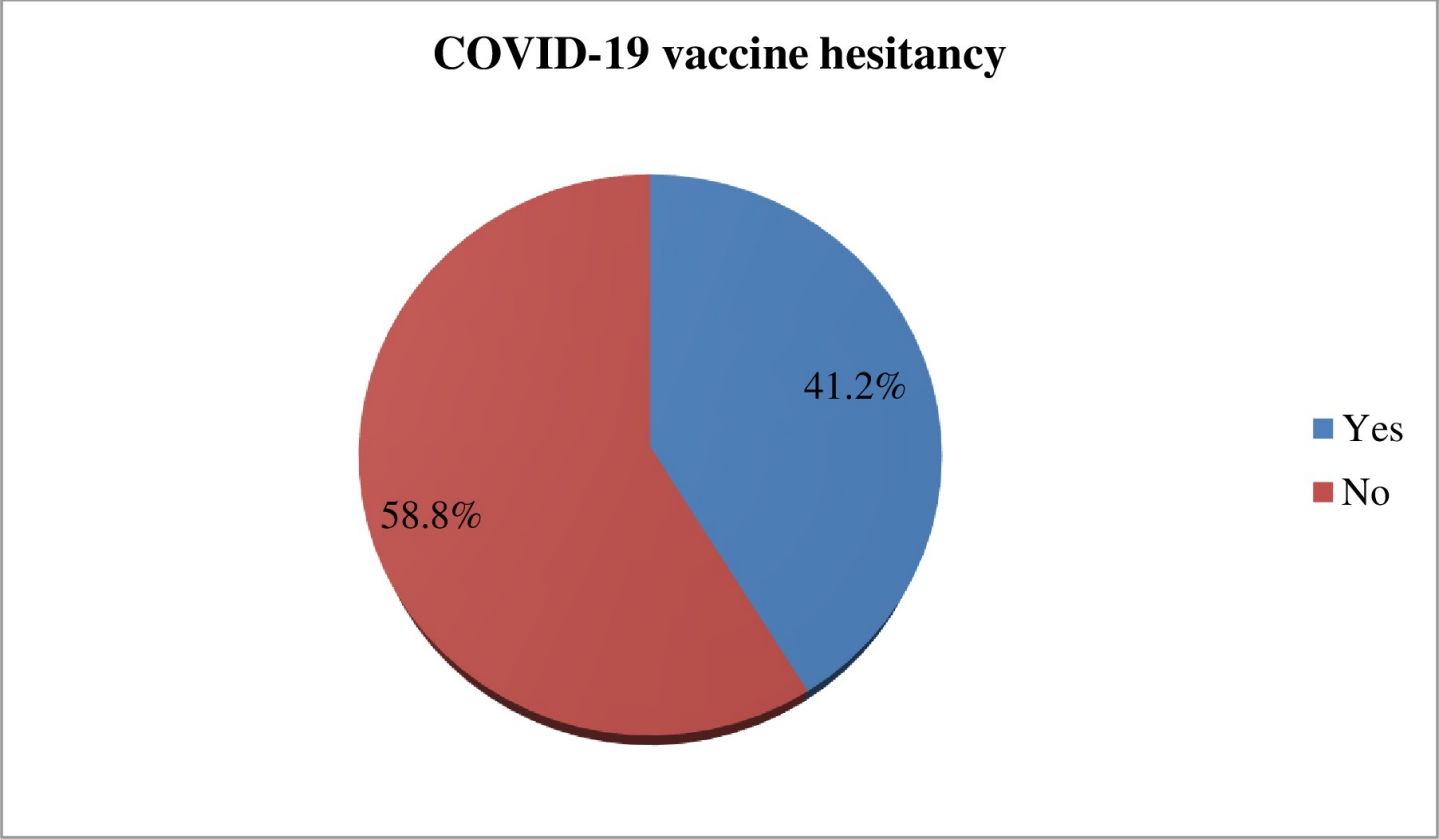
COVID-19 vaccine hesitancy among medical and health science students attending Wolkite University, Ethiopia, 2021 (n = 173).

**Fig 5:**
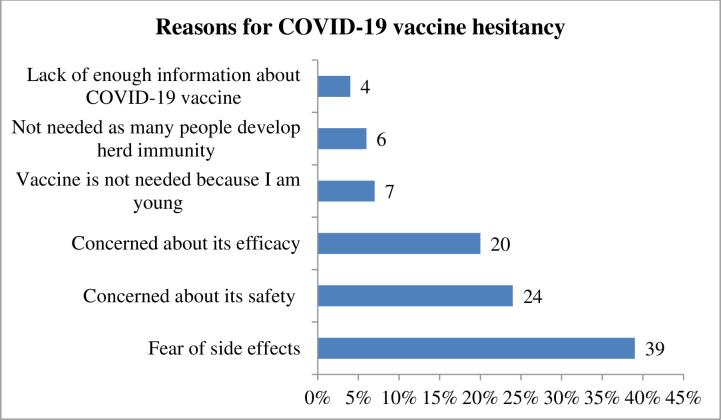
Reasons of COVID-19 vaccine hesitancy among medical and health science students attending Wolkite University, Ethiopia, 2021 (n = 173).

The present study found that 58.8% (95% CI; 53.7%-63.2%) of study participants showed a willingness to receive COVID-19 vaccine if it is once become available. Regarding study participant preference of brand vaccine, 123 (49.8%) of the study participants would prefer Pfizer-BioNTech vaccine followed by AstraZeneca vaccine 81 (32.8%) (Fig 2). The reasons for willingness to receive the COVID‐19 vaccine among those study participants were due to fear of being infected with COVID‐19, 123 (49.7%), followed by fear of infecting my family with COVID‐19, 91(36.8%) (Fig 3). On the other hand, the finding of this study showed that COVID-19 vaccine hesitancy was found to be 41.2% (95% CI; 35.2%, 50.4%) (Fig 4). The major cited reasons for COVID-19 vaccine hesitancy were due to fear of side effects, 68 (39%), concerned about its safety 41 (24%), concerned about its efficacy, 35(20%), not needed as many people develop herd immunity, 11(6%), lack of enough information about COVID-19 vaccine, 6(4%), and vaccine is not needed because I am young, 12 (7%) (Fig 5).

### Factors associated with COVID-19 vaccine hesitancy

On bivariate analysis, age, gender, residence, source of information, and practice towards COVID-19 mitigation measures were significantly associated with COVID-19 vaccine hesitancy. On multivariable logistic regression analysis; student age ≤23 years, being female, rural residence, source of information from social media, and good practice towards the COVID-19 mitigation measures were significantly associated with COVID-19 vaccine hesitancy.

The present study showed that the odds of COVID-19 vaccine hesitancy among medical and health science students age ≤23 years were 1.9 times more likely than those students who were found in the age group >24 years [adjusted odds ratio (aOR) = 1.94, 95% CI; 1.14–3.28]. The odds of COVID-19 vaccine hesitancy among medical and health science students who are female were 1.76 times more likely than those students who are male [aOR = 1.76, 95% CI; 1.14–2.72]. The odds of COVID-19 vaccine hesitancy among medical and health science students who were resided in rural areas were 1.6 times more likely than those students who resided in rural areas [aOR = 1.63, 95% CI; 1.06–2.49]. The odds of COVID-19 vaccine hesitancy among medical and health science students who had received information about the COVID-19 vaccine from social media were approximately 2.7 times more likely than those students who had received information about the COVID-19 vaccine via television/radio [aOR = 2.68, 95% CI; 1.58–4.54]. The odds of COVID-19 vaccine hesitancy among medical and health science students who had good practice with the COVID-19 mitigation measures were 47% less likely than those students who had poor practice [aOR = 0.53, 95% CI; 0.34–0.83] (Table 5).

**Table 5:** Bivariate and multivariate logistic regression analysis of factors associated with COVID-19 vaccine hesitancy among medical and health science students of Wolkite University, Ethiopia, 2021 (n = 420).

Variables		COVID-19 vaccine hesitancy		COR(95% CI)	AOR(95%CI)	P value
		Yes (173)	No (247)			
Age	≤23	146(84.4%)	178(72.1%)	2.09(1.28–3.44)*	1.94(1.14–3.28)*	0.02
	>24	27(15.6%)	69(27.9%)	1	1	
Sex	Female	83(47.9%)	88(35.6%)	1.67(1.12–2.48)*	1.76(1.14–2.72)*	0.01
	Male	90(52%)	159(64.4%)	1	1	
Residency	Rural	109(63%)	118(47.8%)	1.86(1.25–2.77)*	1.63(1.06–2.49)*	0.03
	Urban	64(36.9%)	129(52.2%)	1	1	
Department	Medicine	49(28%)	73(29.6%)	1	1	
	Public health	27(15.6%)	38(15.4%)	0.95(0.51–1.74)	1.15(0.57–2.32)	
	Nursing	51(29.5%)	73(29.6%)	0.96(0.58–1.59)	1.68(0.92–3.08)	
	Medical laboratory	18(10.4%)	31(12.6%)	1.16(0.58–2.29)	1.69(0.77–3.72)	
	Midwifery	28(16.2%)	32(12.9%)	0.77(0.41–1.43)	0.89(0.45–1.79)	
Academic year	2^nd^ year	51(29.5%)	57(23.1%)	0.66(0.3–1.45)	0.71(0.30–1.67)	
	3^rd^ year	46((26.6%)	86(34.8%)	1.11(0.51–2.39)	1.08(0.46–2.49)	
	4^th^ year	63(36.4%)	82(33.2%)	0.77(0.36–1.65)	0.84(0.37–1.92)	
	5^th^ year	13(7.5%)	22(8.9%)	1	1	
Source of information	Social media	48(27.7%)	107(43.3%)	2.06(1.33–3.19)*	2.68(1.58–4.54)*	0.00
	Teachers	11(6.4%)	15(6.1%)	1.26(0.55–2.88)	1.30(0.52–3.25)	
	Family/friends	11(6.4%)	11(4.5%)	0.92(0.38–2.23)	1.01(0.39–2.58)	
	Heath care providers	6(3.5%)	9(3.6%)	1.39(0.48–4.04)	1.22(0.39–3.78)	
	Television/radio	97(56.1%)	105(42.5%)	1	1	
Knowledge	Poor knowledge	29(16.8%)	61(24.7%)	1	1	
	Good knowledge	144(83.2%)	186(75.3%)	1.63(0.99–2.67)	1.58(0.92–2.71)	
Attitude	Poor attitude	27(15.6%)	39(15.8%)	1	1	
	Good attitude	146(84.4%)	208(84.2%)	1.01(0.59–1.73)	0.98(0.54–1.78)	
Practice	Poor practice	114(65.9%)	134(54.3%)	1	1	
	Good practice	59(34%)	113(45.7%)	0.61(0.41–0.92)*	0.53(0.34–0.83)*	0.01

NB. 1 = ref., AOR; Adjusted Odds Ratio, COR; Crude Odds Ratio; CI; Confidence Interval, COVID-19, Corona Viral infectious Diseases 2019.

## Discussion

The present study amid to assess COVID-19 vaccine hesitancy among medical and health students of Wolkite University. Vaccination is one of the promising strategies to halt the transmission of infectious diseases including COVID-19. However, this study found that 42.2% of study participants are hesitant for COVID-19 vaccination when it becomes available. Our finding showed that 78.6% of participants had good knowledge about the COVID-19 vaccine. The finding is higher than studies conducted in Ethiopia (40.8%) [25], Bangladesh (57%) [26], and Egyptians (70.2%) [27]. The possible justification might be due to differences in study period and study population. For instance, our study is conducted among medical and health science students who had awareness regarding the COVID-19 vaccine. However, the study population in Bangladesh and Egyptians are the general population that might lack awareness regarding the COVID-19 vaccine compared to our study participants.

Our study showed that 84.5% of study participants had a positive attitude towards COVID-19 vaccination. The finding is higher than the study conducted in Ethiopia (24.2%) [25] and in Bangladesh (78%) [26]. The possible justification might be medical and health science students might have better awareness regarding vaccine-preventable diseases that might lead them to have a positive attitude towards COVID-19 vaccination.

The present study showed that practice towards COVID-19 mitigation measures was 41%. The finding is lower than a study conducted in Egyptians (49.2%) [27]. However, it is higher than a study conducted in Dirashe district 12.3% [21]. The possible justification might be this study is conducted among medical and health science students who are expected to apply the WHO infection prevention and control protocols during attending class and clinical attachments such as wearing a facemask, utilizing alcohol-based hand sanitizer, and maintaining physical distancing. However, it is lower than a study conducted in Gondar city residents (51.04%) [22]. The possible justification might be study period difference; for instance, the study conducted in Gondar city residents was conducted during the early phase of the COVID-19 pandemic. Thus, there was a mass campaign using mass media platforms to enhance people’s awareness regarding the COVID-19 infection preventive measures that might enable study participants to strictly follow the WHO COVID-19 infection prevention protocols.

COVID-19 vaccine acceptance among medical and health science student was 58.8%. The finding is comparable with a study conducted among United States dental students 56% [28]. On the other hand, the finding is higher than Uganda medical students 37.3% [11], and Ghana 39.3% [29]. However, the finding was lower than undergraduate students from Central and Southern Italy 91.5% [30] and 67% in US [31]. The possible reason for the difference might be the study period difference. For instance, a study in Italy was conducted during the time of COVID-19 vaccine discovery. Therefore, the study participants might easily decide to get vaccinated at a time. However, during the period of our study, a lot of misinformation regarding COVID-19 vaccine was distributed in different social media related to its side effect, ineffectiveness, even it might cause death, and in cumulative such misinformation regarding the vaccine might lead the study participants to hesitate for COVID-19 vaccination.

The most cited reasons for COVID-19 vaccine acceptance are due to fear of being infected with COVID‐19, (49.7%), and fear of infecting my family with COVID‐19, (36.8%). The finding is congruent with a study conducted in Uganda [11] and Egyptian medical students [10]. The finding is supported by a meta-analysis entitled ‘anticipated regret and health behaviour states that despite the availability of the vaccination service, if a person did not take vaccination service and he might get infected with the disease and he transmit it to their friends and family members [32].

Concerning student COVID-19 vaccine brand preference, Pfizer-BioNTech vaccine (49.8%) and AstraZeneca vaccine (32.8%) were chosen by the majority of students. The finding is comparable with studies conducted in Egyptians [10] and Uganda medical students [11]. The possible justification might be the fact that the Pfizer-BioNTech and AstraZeneca vaccine has a higher protection rate compared to the other type of available COVID-19 vaccine.

The magnitude of COVID-19 vaccine hesitancy was 41.2%. The finding is higher than studies conducted among medical students in India 10.6% [12], Uganda 30.7% [11], Italy 13.9% [13], and Michigan medical students 23% [14]. However, the finding is lower than a study conducted in Egyptian medical students (46%) [10] and Southern USA college students 47.5% [24]. The discrepancy of findings might be due to differences in the impact of COVID-19 across countries. For instance, COVID-19 causes more than 4 million deaths in India while in Ethiopia causes 4 thousand deaths. Thus, due to the low risk of death in Ethiopia, they might be hesitant to vaccination. This explanation is supported by a study conducted in 22 countries that reported if individuals perceive that he is at low risk of COVID-19 infection that leads him to a less likely to accept the vaccine [33].

The most commonly cited reason for COVID-19 vaccine hesitancy is due to fear of side effects, 39%, concerned about its safety, 24%, concerned about its efficacy, 20%. The finding is in agreement with studies conducted in Addis Ababa [34], Uganda [11], Egyptians [10], India [12], Saudi Arabia [35], and China [36]. The possible justification might be due to the vaccine development period were not taken the adequate time that might affect its effectiveness, and the competition of the countries to discover the vaccine and to control the economy might lead them to the unproductive finding regarding the vaccine, thus it might create fear to the study participants.

The present study showed that the odds of COVID-19 vaccine hesitancy among medical and health science students ≤23 years were 1.9 times more likely than those students >24 years. The finding is comparable with a study conducted in Portugal [37]. The possible justification might be those students who were found ≤23 years old might consider themselves as healthy, young, and have immunity that could protect them from severe COVID-19 complications.

The odds of COVID-19 vaccine hesitancy among medical and health science students who are female were 1.76 times more likely than those students who are male. The finding is comparable with a study conducted among medical students in Uganda [11] and Portugal [37]. The possible justification might be due to the distribution of misinformation and conspiracies theories regarding the COVID-19 vaccine might cause menstrual disturbance and infertility [38].

The odds of COVID-19 vaccine hesitancy among medical and health science students who was resided in rural area were 1.6 times more likely than those students resided in rural areas. The finding is comparable with a study conducted in Bangladesh adults [39]. The possible justification might be those who were lived in rural areas might lack up-to-date information regarding the safety and effectiveness of COVID-19 vaccine as compared to those who were resided in urban areas.

The present study showed that the odds of COVID-19 vaccine hesitancy among medical and health science students who had received information about the COVID-19 vaccine from social media were approximately 2.7 times more likely than those students who had received information from mass media. The finding is comparable with a study conducted in Addis Ababa [34] and India [12]. For instance, a study conducted in Addis Ababa shows that those who had received information regarding the COVID-19 vaccine from social media were approximately 3.6 times more likely hesitant for COVID-19 vaccination. The possible justification might be social media such as Facebook and Telegram might distribute misinformation regarding the available COVID-19 vaccine that might negatively influence student willingness to receive COVID-19 vaccination. It is, therefore, crucial that student’s sources of information should rely on mass media platforms rather than social media.

The odds of COVID-19 vaccine hesitancy among students who had good practice towards COVID-19 mitigation strategies were 47% less likely than those students who had poor practice. The possible justification might be those students who had good practice to COVID-19 mitigation measures might have awareness regarding COVID-19 prevention protocols and the available COVID-19 vaccine which might lead them to be less likely to be hesitant from receiving COVID-19 vaccination.

### Limitation of study

The present study was not void of limitation. This study lack qualitative data supplementation to explore socio-cultural barriers of vaccine hesitancy among medical and health science students.

### Conclusion and recommendation

COVID-19 vaccine hesitancy is found to be high. Therefore, students are advised to receive COVID-19 vaccine information from government lead mass media (i.e. television and radio), increase student awareness, and adherence to COVID-19 mitigation measures is recommended. Moreover, before vaccine campaign policy makers and stakeholders should work on the identified factors to enhance vaccine acceptance in the study area and to control the pandemic.

## Supporting information

S1 File English version questionnaires. (DOCX)

S2 File Minimal data set. (SAV)

## Abbreviation

COVID-19: Corona Virus Infectious Diseases 2019
aOR: adjusted Odds Ratio
WHO: World Health Organization
