# Supplementary material for: COVID-19 vaccine hesitancy among medical and health science students attending Wolkite University in Ethiopia
Source: PLoS One. 2022 Jan 25;17(1):e0263081. doi: 10.1371/journal.pone.0263081 (PMC8789154; doi:10.1371/journal.pone.0263081)
Supplement: S1 File — (DOCX) [file pone.0263081.s001.docx]

**English Version Questionnaires**

**Questionnaires to assess COVID-19 vaccine hesitancy among medical and health science students at Wolkite University, 2021**

| **Part one: socio-demographic characteristics** | | | | | | |
| --- | --- | --- | --- | --- | --- | --- |
| 1. Gender | 1. Female 2.Male | | | | | |
| 1. Age | 1. 19-23 2.24-28 | | | | | |
| 1. Residence | 1. Rural 2.Urban | | | | | |
| 1. Marital status | 1. Single 2/Married /engaged | | | | | |
| 1. Mother educational status | 1/No formal education 2/Primary education 3/Secondary and above | | | | | |
| 1. Father educational status | 1/No formal education 2/Primary 3/Secondary and above | | | | | |
| 1. Department | 1/Medicine 2/Public health 3/Midwifery 4/Nursing 5/Medical laboratory | | | | | |
| 1. Academic year | 1. 2nd year 2/3rd year 3/4th year 4/5th year | | | | | |
| **Part two: knowledge related items** | | | | | | |
| 1. COVID-19 vaccine is available in Ethiopia | | | | | 1/Yes 2/No 3/I don’t know | |
| 1. COVID-19 vaccine is effective | | | | | 1/Yes 2/No 3/I don’t know | |
| 1. COVID-19 vaccine should be given for HCWs firstly | | | | | 1/Yes 2/No 3/I don’t know | |
| 1. COVID-19 vaccination is important for overall public health | | | | | 1/Yes 2/No 3/I don’t know | |
| 1. COVID-19 vaccination doesn’t cause autoimmune diseases | | | | | 1/Yes 2/No 3/I don’t know | |
| 1. COVID-19 vaccination doesn’t cause allergic reaction | | | | | 1/Yes 2/No 3/I don’t know | |
| 1. COVID-19 vaccination decrease severity of diseases | | | | | 1/Yes 2/No 3/I don’t know | |
| **Part three: Attitude towards COVID-19 infection** | | | | | | |
| 1. COVID-19 vaccine should be made mandatory for the health care workers? | | | | 1. Agree | | |
|  |  |  |  | 1. Disagree | | |
|  |  |  |  | 1. I am not Shure | | |
| 1. COVID-19 vaccination should be mandatory for the general public | | | | 1. Agree | | |
|  |  |  |  | 1. Disagree | | |
|  |  |  |  | 1. I am not Shure | | |
| 1. I will take the COVID-19 vaccine; if it is available in the hospital right know | | | | 1. Agree | | |
|  |  |  |  | 1. Disagree | | |
|  |  |  |  | 1. I am not Shure | | |
| 1. COVID-19 vaccine reduce the spread of the disease in the community | | | | 1. Agree | | |
|  |  |  |  | 1. Disagree | | |
|  |  |  |  | 1. I am not Shure | | |
| 1. I will encourage my family/friends/relatives to get vaccinated? | | | | 1. Agree | | |
|  |  |  |  | 1. Disagree | | |
|  |  |  |  | 1. I am not Shure | | |
| 1. COVID-19 vaccine is safe | | | | 1. Agree | | |
|  |  |  |  | 1. Disagree | | |
|  |  |  |  | 1. I am not Shure | | |
| 1. COVID-19 vaccination reduces the severity of the disease? | | | | 1. Agree | | |
|  |  |  |  | 1. Disagree | | |
|  |  |  |  | 1. I am not Shure | | |
| Part four: practice related questionnaires | | | | | | |
| 1. Frequent utilization of face mask | | | | | | 1. Yes 2/No |
| 1. Maintained physical distancing at least 6 feet (2m) | | | | | | 1. Yes 2/No |
| 1. Covered your mouth during coughing /sneezing/ | | | | | | 1. Yes 2/No |
| 1. Avoid greeting with handshaking, hanging/cheek kissing | | | | | | 1. Yes 2/No |
| 1. Frequent utilized alcohol-based hand sanitizer | | | | | | 1. Yes 2/No |
| 1. Regular hand washing using water and soap | | | | | | 1. Yes 2/No |
| 1. Avoided touching face (eyes, nose, mouth) | | | | | | 1. Yes 2/No |
| 1. Clean and disinfecting frequently touched objects like phone | | | | | | 1. Yes 2/No |
| 1. Stay at home when having flu-like symptoms | | | | | | 1. Yes 2/No |
| 1. Avoid going unnecessarily to crowed place | | | | | | 1. Yes 2/No |
| 1. Avoid gathering with many people | | | | | | 1. Yes 2/No |
| 1. Avoid eating uncooked food | | | | | | 1. Yes 2/No |
| Part 5: COVID-19 vaccine related questionnaires | | | | | | |
| 1. ‘‘Do you have an intention to be vaccinated against COVID-19 infection, if the COVID-19 vaccine is available right now?’’ | | | 1. Yes 2. No | | | |
| 1. If **yes for q no 1** what is your Motivations behind receiving COVID-19 vaccine | | | 1. Fear of being infected with COVID‐19 2. Fear of infecting my family with COVID‐19 3. Belief in the effectiveness and safety of the vaccine 4. Availability of free vaccines 5. Others | | | |
| 1. If **yes for q no 1** what type of COVID-19 vaccine brand you prefer to be vaccinated | | | 1. Pfizer-BioNTech vaccine 2. AstraZeneca vaccine 3. Moderna vaccine 4. Janssen vaccine 5. Any brand | | | |
| 1. If the answer is **no** **for question number one** what is the reason for not vaccinated | | 1. Fear of side effects 2. Concerned about its safety 3. Concerned about its efficacy 4. Vaccine is not needed because I am young 5. Not needed as many people develop herd immunity 6. Lack of enough information about COVID-19 vaccine | | | | |
| 1. Source of information about COVID-19 vaccine | | 1. Mass media (television/radio) 2. Health care workers 3. Teachers 4. Family/friends 5. Social media Facebook/ television | | | | |

**Thank you Participant signature …………….**
